# An unruptured posterior communicating artery aneurysm ruptured during angiography: A case report

**DOI:** 10.1097/MD.0000000000017785

**Published:** 2019-11-01

**Authors:** Songtao Guo, Xi Wu

**Affiliations:** Department of Neurosurgery, Xinganmeng People Hospital, Ulanhot, China.

**Keywords:** aneurysm rupture, angiography, oculomotor nerve palsy, posterior communicating artery aneurysm

## Abstract

**Introduction:**

Cerebrovascular imaging is the gold standard for diagnosis of intracranial aneurysms. Rupture of intracranial aneurysm is rare in cerebrovascular angiography, especially in unruptured intracranial aneurysm.

**Patient concerns:**

A 74-year-old woman was admitted to the hospital for sudden onset of left eyelid ptosis for 1 day with no obvious inducement. The patient had a history of hypertension. Physical examination revealed that she had clear consciousness and normal speech, but the left eyelid drooped. The left pupil diameter was 5 mm and light reflex was absent. The left eyeball could not move, and the right eye examinations were normal. The limb muscle strength and muscle tension were normal.

**Diagnosis:**

Bilateral internal carotid artery posterior communicating aneurysm, severe stenosis of the origin of left carotid artery, and right oculomotor nerve palsy.

**Interventions:**

After the hospital, the aneurysm ruptured and hemorrhaged during radiography, and the patient improved after immediate rescue and treatment. On the third day after angiography, the patient's the condition gradually stabilized. Under the general anesthesia, left carotid artery stenosis stent implantation and left posterior communicating artery aneurysm stent assisted coil embolization were performed successfully. On the second day after embolization, the patient's head computed tomography (CT) showed subarachnoid hemorrhage with hydrocephalus. The patient underwent external ventricular drainage. A month later, the patient underwent ventriculoperitoneal shunt.

**Outcomes:**

Six months later, the patient visited our hospital for a follow-up, and she was clear-headed, aphasia, right limb hemiplegia with muscle strength grade II, left side autonomous activities, and the GOS score was 2 points. Head CT showed the ventricles were normal.

**Conclusions:**

Acute oculomotor palsy may be a risk factor for rupture of ipsilateral unruptured aneurysms, but more basic research and clinical trial evidence of intracranial aneurysms are needed to confirm this.

## 1 Introduction

Intracranial aneurysms are usually abnormal bulges on the wall of intracranial arteries and the first cause of subarachnoid hemorrhage. In recent years, with the popularization of the imaging techniques, such as digital subtraction angiography, computed tomography angiography (CTA), and magnetic resonance angiography, the clinical detection rate of asymptomatic unruptured intracerebral aneurysms (UIAs) has been improved. The incidence of UIAs ranged from 5% to 8%.^[1,2]^ The rupture rate of UIAs is low, but the prognosis of ruptured UIAs is poor. About 30% of the patients died outside the hospital, whereas about 15% of the patients who received treatment in hospital died, and most of the survivors have sequela of hemiplegia, aphasia, and other functional disorders.^[1]^ Morphology and hemodynamics of aneurysms are important factors affecting rupture of aneurysms.^[3–5]^ Posterior communicating artery aneurysms (PCAs) are the common site of aneurysms, accounting for about 45.9% of all aneurysms,^[6]^ and have a high rupture rate.^[2]^ Oculomotor nerve palsy (ONP) is a common clinical manifestation of PCAs because of the adjacent anatomical relationship.^[7]^ About 30% to 50% of patients with PCAs will develop ONP.^[8–10]^ Commonly, the aneurysms with a diameter greater than 4 mm^[11]^ or 7 cm^[10]^ will cause ONP. Cerebral angiography is the gold standard for the diagnosis of intracranial aneurysms.

During the procedure of cerebral angiography, rupture of intracranial aneurysms is rare, and rupture of UIAs is even more rare. Here, we reported a case of posterior communicating artery UIA with ONP as the initial clinical manifestation, and the aneurysm ruptured during the angiography. The patient has provided written informed consent for publication of the case. This study was approved by the Ethics Committee of XinAnMeng Hospital.

## 2 Case presentation

**Figure 1:**
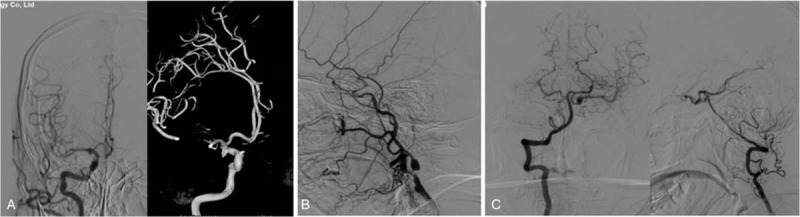
Carotid angiogram of the patient. (A) Right carotid angiography revealed right posterior communicating artery aneurysm. (B) Left carotid artery stenosis, and decreased blood flow. (C) Left vertebral arteriography for posterior communicating artery aneurysms.

**Figure 2:**
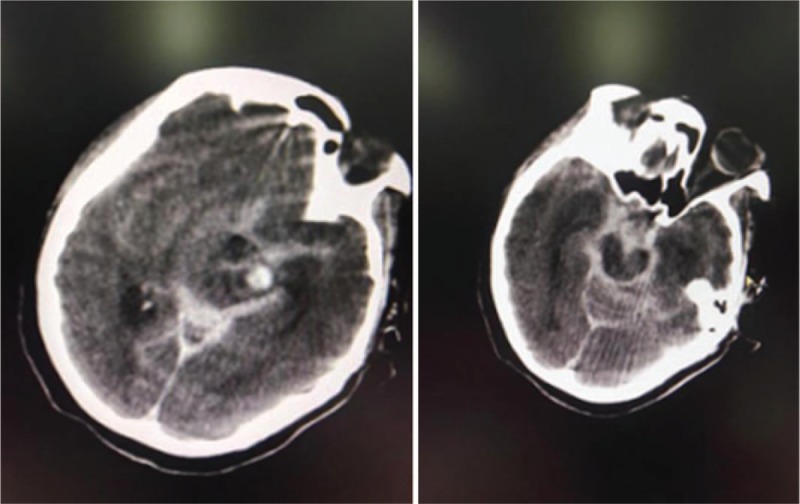
Emergency CT examination of the patient during the cerebral angiography. The images of CT showed ruptured aneurysm. CT = computed tomography.

A 74-year-old woman was admitted to the hospital for sudden onset of left eyelid ptosis for 1 day with no obvious inducement. The patient had a history of hypertension, but she did not regularly measure blood pressure and take oral antihypertensive drugs. Physical examination revealed that she had clear consciousness and normal speech, but the left eyelid drooped. The left pupil diameter was 5 mm and light reflex was absent. The left eyeball could not move, and the right eye examinations were normal. The limb muscle strength and muscle tension were normal. CTA examination showed bilateral internal carotid artery aneurysms. After admission, the whole cerebral angiography was performed under local anesthesia and the procedure was smooth. The imaging diagnosis was bilateral carotid artery communicating aneurysm combined with severe stenosis of the origin of left carotid artery. The size of the left aneurysm was about 8 mm × 3 mm and the neck was about 3 mm, and the right aneurysm was about 3.7mm × 3.8 mm and the neck was about 3.8 mm (Fig. 1). After the operation, when the patient was ready to be lifted down the bed after the angiography, she suddenly became unconscious and could not open her eyes. We found bilateral pupil dilation with a diameter of 5 mm, absence of the light reflex, and there was no reaction to the stabbing pain in the limbs. The patient's emergency head computed tomography (CT) examination showed subarachnoid hemorrhage (Fig. 2). The doctor immediately gave the patient intravenous injection of 20% mannitol solution 250 mL. About 15 minutes later, the patient regained consciousness, questions could be answered, and the limbs could move autonomously. We considered the possibility of rupture of the left posterior communicating artery aneurysm. Because of her older age and poor physical general condition of the body, the main worry about the patient was that she might show a poor tolerance to operation and anesthesia. Thus, the patient received conservative treatment.

**Figure 3:**
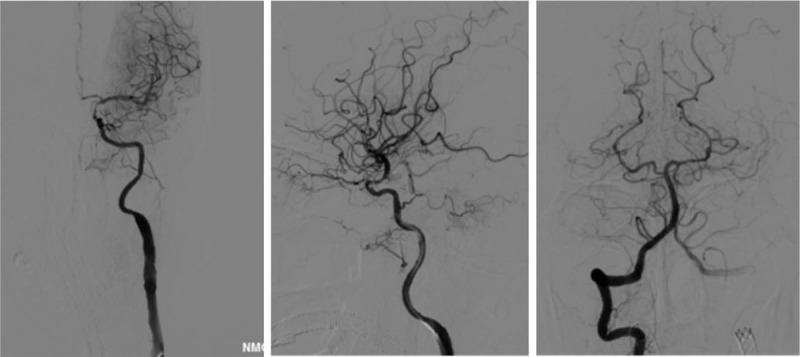
Left carotid artery stenosis stent implantation and left posterior communicating artery aneurysm stent assisted coil embolization were performed successfully.

**Figure 4:**
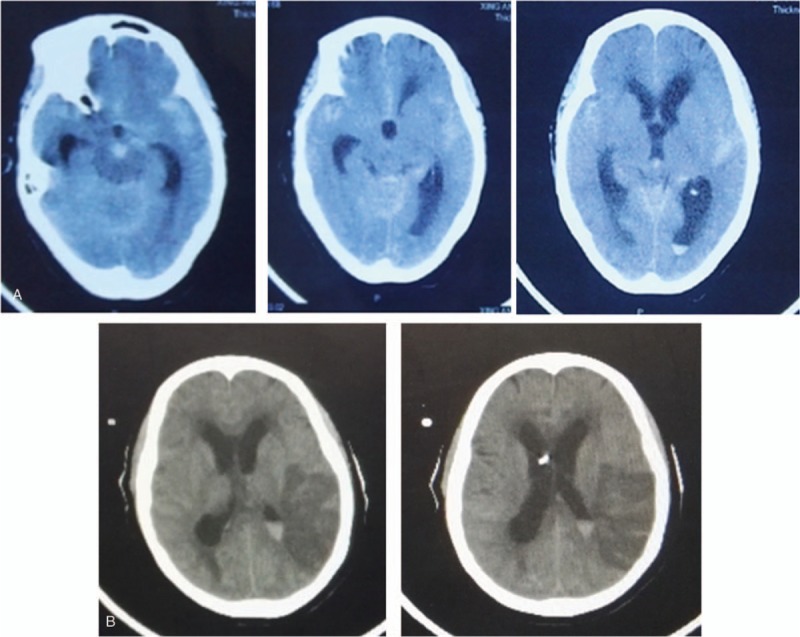
Emergency CT examination of the patient after 3 days of cerebral angiography. The images of CT showed hydrocephalus (A), an emergency external ventricular drainage was performed. Postoperative CT scan showed cerebral infarction in the left temporal lobe (B). CT = computed tomography.

**Figure 5:**
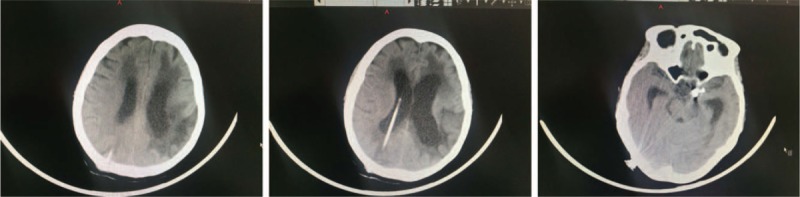
Head CT showed relief of hydrocephalus after ventriculoperitoneal surgery. CT = computed tomography.

On the third day after operation, the patient's condition gradually improved. Under the general anesthesia, left carotid artery stenosis stent implantation and left posterior communicating artery aneurysm stent assisted coil embolization were performed successfully (Fig. 3). On the second day after embolization, the patient was in a coma with grade 0 muscle strength on the right limb, positive on the right babinski, and occasional voluntary movement on the left limb. Head CT showed subarachnoid hemorrhage with hydrocephalus (Fig. 4A). Day after surgery, emergency external drainage was performed, and the postoperative CT scan showed cerebral infarction in the left temporal lobe (Fig. 4B). Postoperative 250 mL of normal saline + 30 mL of edaravone injection was injected twice a day, 50 mL of normal saline + 24 mg of nimodipine injection was pumped continuously, and 250 mL of normal saline + 40 mg of monosialotetrahexosylganglioside sodium injection were injected once a day. The patient's condition was stable. A month later, the patient underwent ventriculoperitoneal shunt. The operation was successful and the postoperative condition improved. Six months later, the patient visited our hospital for a follow-up, and she was clear-headed, aphasia, right limb hemiplegia with muscle strength grade II, left side autonomous activities, and the GOS score was 2 points. Head CT showed the ventricles were normal (Fig. 5).

## 3 Discussion

It is reported that the incidence of intracranial aneurysm rupture in cerebral angiography is 0.01% to 0.35%.^[12]^ In a report in 5484 patients with subarachnoid hemorrhage, 7 patients had ruptured intracranial aneurysms,^[13]^ and this is similar to the results reported by Aoyagi and Hayakawa.^[12]^ In 1984, Tsementzis et al^[14]^ reported 3 cases of intracranial aneurysms ruptured again during angiography. All patients died, and 2 of them were posterior communicating artery aneurysms. In 1995, Saitoh et al^[15]^ report 144 cases of subarachnoid hemorrhage caused by intracranial aneurysms in patients with cerebral angiography and 2 cases of ruptured hemorrhage, accounting for 1.4%. In 1999, a case of rupture of posterior inferior cerebellar aneurysm during angiography was first reported by Gailloud and Murphy,^[16]^ but not in detail. In 2002, Zaehringer et al^[17]^ reported 2 cases of aneurysm rupture during cerebral angiography, and the prognosis of the patients were poor. Liu et al^[18]^ in 2003 reported 3 cases of ruptured intracranial aneurysms during cerebral angiography. All of them were treated with clipping craniotomy. No death occurred after operation, but the prognosis was also poor. Wang and Zhao^[19]^ reported 8 cases of rupture of intracranial arteriography. All patients died after operation, and 2 of them were UIAs.

There are many reasons that influence the rupture in angiography of the unruptured aneurysm. Once it occurs, the prognosis of the patients becomes very poor. Therefore, it is of great clinical significance to study the clinical characteristics of UIA-ruptured patients. In recent years, many scholars have been looking for a method to predict the rupture of unruptured aneurysms. Although many new techniques and viewpoints have emerged, they cannot completely predict the actual situation in clinical practice. It is generally believed that it is related to the location and shape of the aneurysm, and also the timing and pressure of the imaging.^[15,20,21]^ The most important is the size of aneurysms. Both International Study of Unruptured Intracranial Aneurysms^[22]^ and Unruptured Cerebral Aneurysm Study^[23]^ regard diameter as an important factor in evaluating rupture of an UIA. These 2 studies also suggest a higher risk of rupture of internal carotid artery aneurysms and posterior communicating artery aneurysms. The case in this study was a posterior communicating artery aneurysm with a diameter greater than 7 mm with high risk of rupture.^[22]^ In addition, intracranial aneurysms are 1 of the main causes of ONP,^[24]^ and accounts for about 36% of nontraumatic ONP, especially in acute unilateral ONP.^[25]^ Among them, the posterior communicating artery aneurysms are most common.^[26–28]^ The mechanism mainly includes the space-occupying pressure of the aneurysm and its pulsatile stimulation.^[29]^ In this report, acute ipsilateral ONP was the first symptom of the patient, and the possibility of sudden aneurysm enlargement could not be ruled out. We suspected that the thickness and toughness of local walls of aneurysms might be poor. Finally, during the angiographic process, the posterior communicating artery aneurysm was not well displayed after left carotid angiography, and the left posterior communicating artery aneurysm was clearly displayed after left vertebral angiography. It indicates that the blood flow of posterior communicating aneurysms mainly originates from the posterior circulation rather than the left internal carotid artery. Suddenly, increased intracranial aneurysm blood flow and pressure during posterior circulation angiography continuous injection of contrast medium with high-pressure syringe might be another main cause of ruptured posterior communicating artery aneurysm hemorrhage. In conclusion, the causes of rupture of unruptured aneurysm in cerebral angiography may be as follows: large aneurysm size and irregular shape; oculomotor nerve palsy indicates the possibility of acute enlargement of aneurysm, increasing the risk of rupture; and aneurysm blood flow and pressure increase sharply during angiography.

## 4 Conclusions

We reported a UIA patient with acute unilateral ONP as an initial clinical manifestation. Clinicians should pay attention to such case because of the high possibility of rupture of the aneurysm and poor prognosis of ruptured posterior communicating artery aneurysm. The limitation of this study, however, was that a sudden increase in the size of aneurysms causing the acute ONP of the patient was only our clinical judgment, and lack of comparison of angiographic images of patients before the ONP. However, more studies are needed to determine whether acute oculomotor nerve palsy can be a risk factor for rupture of ipsilateral unruptured communicating aneurysm.

## Author contributions

**Conceptualization:** Xi Wu.

**Project administration:** Songtao Guo.

**Supervision:** Xi Wu.

**Writing – original draft:** Songtao Guo, Xi Wu.

**Writing – review & editing:** Songtao Guo, Xi Wu.
